# Clinical impact of ampulla of Vater cancer subtype classification based on immunohistochemical staining

**DOI:** 10.1186/s12957-023-03289-y

**Published:** 2024-01-03

**Authors:** Chae Hwa Kwon, Ji Hyun Ahn, Hyung Il Seo, Dong Uk Kim, Sung Yong Han, Suk Kim, Nam Kyung Lee, Seung Baek Hong, Young Mok Park, Byeong Gwan Noh

**Affiliations:** 1 Biomedical Research Institute, Pusan National University Hospital, Busan, South Korea; 2 Department of Pathology, Pusan National University School of Medicine and Biomedical Research Institute, Pusan National University Hospital, Busan, South Korea; 3 Department of Surgery, Pusan National University School of Medicine and Biomedical Research Institute, Pusan National University Hospital, 179 Gudeok-Ro, Seo-Gu, Busan, 49241 South Korea; 4 Department of Internal Medicine, Pusan National University School of Medicine and Biomedical Research Institute, Pusan National University Hospital, Busan, South Korea; 5 Department of Radiology, Pusan National University School of Medicine and Biomedical Research Institute, Pusan National University Hospital, Busan, South Korea

**Keywords:** ampullar of Vater cancer, Histological subtypes, Immunohistochemical staining, CK7, CDX2, Disease-free survival

## Abstract

**Background:**

The histological subtype is an important prognostic factor for ampulla of Vater (AoV) cancer. This study proposes a classification system for the histological subtyping of AoV cancer based on immunohistochemical (IHC) staining and its prognostic significance.

**Methods:**

Seventy-five AoV cancers were analyzed for cytokeratin 7 (CK7), CK20, and causal-type homeobox transcription factor 2 (CDX2) expression by IHC staining. We differentiated the subtypes (INT, intestinal; PB, pancreatobiliary; MIX, mixed; NOS, not otherwise specified) into classification I: CK7/CK20, classification II: CK7/CK20 or CDX2, classification III: CK7/CDX2 and examined their associations with clinicopathological factors.

**Results:**

Classifications I, II, and III subtypes were INT (7, 10, and 10 cases), PB (43, 37, and 38 cases), MIX (13, 19, and 18 cases), and NOS (12, 9, and 9 cases). Significant differences in disease-free survival among the subtypes were observed in classifications II and III using CDX2; the PB and NOS subtype exhibited shorter survival time compared with INT subtype. In classification III, an association was revealed between advanced T/N stage, poor differentiation, lymphovascular invasion (LVI), the PB and NOS subtypes, and recurrence risk. In classification III, the subtypes differed significantly in T/N stage and LVI. Patients with the PB subtype had advanced T and N stages and a higher incidence of LVI.

**Conclusions:**

Classification using CDX2 revealed subtypes with distinct prognostic significance. Combining CK7 and CDX2 or adding CDX2 to CK7/CK20 is useful for distinguishing subtypes, predicting disease outcomes, and impacting the clinical management of patients with AoV cancer.

**Supplementary Information:**

The online version contains supplementary material available at 10.1186/s12957-023-03289-y.

## Background

Ampulla of Vater (AoV) cancer is extremely rare, accounting for 0.2–0.6% of gastrointestinal cancers and 6% of periampullary cancers, which are malignant tumors occurring within 2 cm of the duodenal ampulla [1–3]. Despite its rarity, the incidence of AoV cancer has increased worldwide. Fortunately, owing to the relatively early detection of periampullary cancers, the resection rate is high, resulting in a favorable prognosis. However, the five-year survival rate is approximately 40–60% [4, 5].

Prognostic factors for AoV cancer include histologic type, advanced T stage, lymph node metastases, R0 resection, histological differentiation, and lymphovascular invasion (LVI) [6–8]. Histological subtype has emerged as a significant prognostic factor, suggesting the need for distinct adjuvant treatment approaches [9–13]. AoV cancers are primarily categorized into two histological subtypes. The intestinal (INT) type originates from the duodenal mucosa and exhibits characteristics similar to duodenal cancers. The pancreatobiliary (PB) type is an adenocarcinoma originating from the ductal endothelium (the bile or pancreatic duct) and shares similarities with distal bile duct cancer or pancreatic cancer [14, 15]. Thus, the PB type is generally associated with a poor prognosis [9–11].

The interpretation of morphological features in AoV cancer varies among pathologists, resulting in a reading match rate of approximately 50–77% [15, 16]. Additionally, the presence of a significant proportion of mixed types, encompassing the INT and PB subtypes, poses challenges in determining the dominant subtype. In view of this, various immunohistochemical (IHC) stains have been employed to address the subjectivity of morphological assessments [15–18]. In the case of the INT type AoV cancer, there is often expression of cytokeratin 20 (CK20), whereas the PB type typically exhibits cytokeratin 7 (CK7) expression. Causal-type homeobox transcription factor 2 (CDX2) has been reported to show high sensitivity and specificity for the INT subtype [17, 18]. However, the morphological features and IHC results do not always align perfectly. Despite this, IHC provides a quantitative analysis that offers a more objective approach than the qualitative assessment of morphological features [15–18].

Therefore, this study aimed to propose a simpler and more objective classification of AoV subtypes by relying solely on IHC staining for CK7, CK20, and CDX2 expression and interpreting the clinical implications.

## Methods

### Study design and patient selection

The study period extended from January 2008 to December 2019 and 75 patients who underwent curative-intent pancreaticoduodenectomy resection for AoV adenocarcinoma were used for the study. Furthermore, the postoperative surgical epecimens’ were subjected to histological examination and subsequent CK7/CK20/CDX2 IHC testing were included in the study. These patients were regularly followed up every 6 months until recurrence. The patients with R1/R2 resections, stage IV disease, or morphological subtypes described solely without IHC staining, as well as patients with other adenocarcinomas, such as signet ring cell type, mucinous carcinoma, neuroendocrine carcinoma, and sarcoma, were excluded. This retrospective study was approved by the Institutional Review Board of the Clinical Trial Center in our hospital with registration number (IRB No. 2303–007-124), and written informed consent was obtained from all participants. The clinical information from patient records was reviewed retrospectively.

### Follow-up and survival estimation

The patient’s follow-up was performed twice yearly which involved the use of abdominal and thoracic computed tomography scans and tumor marker detection (CA19-9 and CEA). Each patient's medical history was used to determine the differences between disease-free survival (DFS) and recurrence based on imaging studies. The overall survival (OS) rate was calculated from the date of surgery to the patient's death or until March 31, 2023, using medical records or public administration data.

### IHC staining

Immunohistochemistry for CK7 and CK20 was performed using a fully automated Bond Max automatic slide staining machine (Leica Microsystems, Bannockburn, IL, USA) on formalin-fixed paraffin-embedded tissue sections. The monoclonal antibody clones against CK7 (OV-TL12/30, 1:2,000), CK20 (PW31, 1:100), and CDX2 (EPR2764Y, 1:50) were diluted and used. The immunoreactivity grade for CK7/CK20/CDX2 marker expression was assessed based on the percentage of positive cells (0, no cell expression; 1, tumor cells < 25%; 2, tumor cells 25–50%; and 3, tumor cells > 50%). IHC positivity was defined as grade 3 or 4 tumor staining. The IHC results were further subdivided into intestinal (INT), pancreatobiliary (PB), mixed (MIX), and not otherwise specified (NOS) subtypes based on the combination of these markers.

### Classification of subtypes according to IHC staining

The subtypes were classified into the following three categories based on positivity for CK7, CK20, and CDX2 expression: classification I (INT type, CK7-/CK20 + and CDX2 + ; PB type, CK7 + /CK20- and CDX2-; MIX type, CK7 + /CK20 + or CDX2 + ; NOS type, CK7-/CK20 + or CDX2 +); classification II (INT type, CK7-/CK20 + or CDX2 + ; PB type, CK7 + /CK20- or CDX2-; MIX type, CK7 + /CK20 + or CDX2 + ; NOS type, CK7-/CK20- or CDX2-); and classification III (INT type, CK7-/CDX2 + ; PB type, CK7 + /CDX2-; MIX type, CK7 + /CDX2 + ; NOS type, CK7-/CDX2-). Supplementary Fig. 1 shows the IHC images.

### Statistical analysis

The clinicopathological characteristics of patients with AoV cancer with different subtypes were compared and analyzed using the Mann–Whitney U and chi-square tests for continuous and categorical variables, respectively. Kaplan–Meier survival analyses with the log-rank test were used to compare the survival of patients with different subtypes. Univariate and multivariate analyses were performed using Cox proportional hazard models. Statistical analyses were performed using the R software (version 4.2.1). The R packages “moonBook,” “survminer,” and “survival” were used. Statistical significance was set at *P* < 0.05.

## Results

### Subtype classification of AoV cancer based on IHC staining

AoV cancer subtypes were classified based on CK7, CK20, or CDX2 expression as determined by IHC staining (Table 1). In classification I, subtypes were determined based on CK7 and CK20 expression. The INT subtype had 7 cases with CK7-/CK20 + expression, and the PB subtype had 43 cases with CK7 + /CK20- expression. The MIX subtype included 13 cases with CK7 + /CK20 + expression, and the NOS subtype included 12 cases with CK7-/CK20- expression. Using CDX2 for subtyping, CK7, CK20, or CDX2 expressions were used for classification II, and CK7 and CDX2 expressions were used for classification III. The INT subtype was consistent in classifications II and III for CK7-/ CDX2 + or CK20 + expression (10 cases), and the NOS subtype remained the same in classifications II and III for CK7-/CK20-/CDX2- expression (9 cases). Among the 19 cases with CK7-, 10 cases with CDX2 + included 7 cases with CK20 + . The PB and MIX subtypes were identified differently in classifications II (37 cases with CK7 + /CK20-/CDX2- and 19 cases with CK7 + , CK20 + , or CDX2 + , respectively) and III (38 cases with CK7 + /CDX2- and 18 cases with CK7 + /CDX2 + , respectively). Among the 56 cases with CK7 positivity, 18 cases with CDX2 positivity included 12 cases with CK20 positivity except for one case.

**Table 1:** Classification of AoV cancer subtype based on immunohistochemical staining of CK7, CK20, and CDX2

CK7		CK20		CDX2		Classification I	Classification II	Classification III
Positivity	n	Positivity	n	Positivity	n			
-	19	-	12	-	9	NOS	NOS	NOS
				+	3		INT	INT
		+	7	-	0	INT	INT	NOS
				+	7		INT	INT
+	56	-	43	-	37	PB	PB	PB
				+	6		MIX	MIX
		+	13	-	1	MIX	MIX	PB
				+	12		MIX	MIX

*AoV* Ampullar of Vater, *INT* Intestinal subtype, *MIX* Mixed subtype, *NOS* Not otherwise specified, *PB* pancreatobiliary subtype

### Survival analysis according to AoV cancer subtype classification

**Fig. 1:**
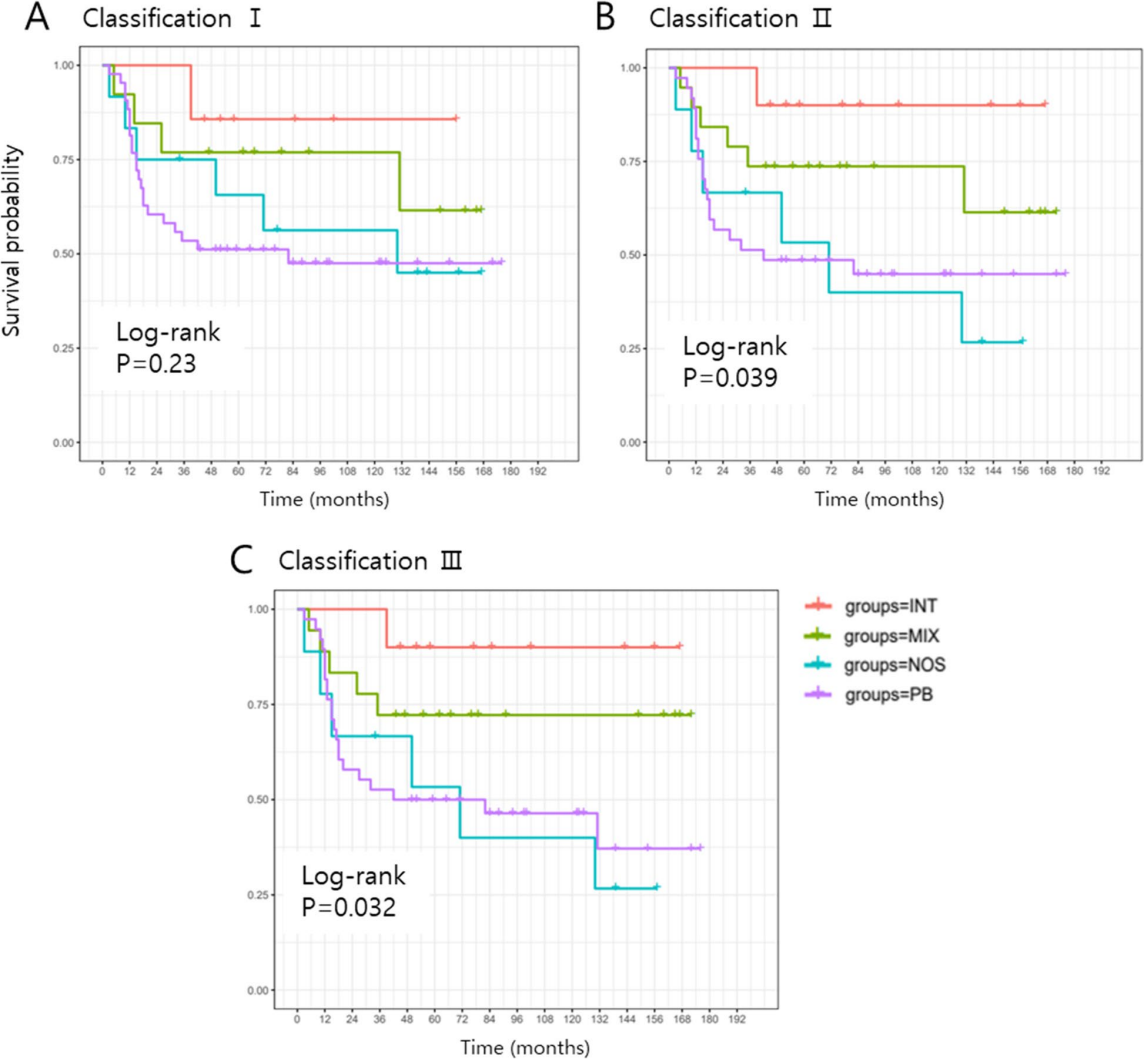
Kaplan–Meier curves for disease-free survival of patients with AoV cancer according to subtype classifications. **A** Classification I. **B** Classification II. **C** Classification III. AoV, ampullar of Vater; INT, intestinal subtype; MIX, mixed subtype; NOS, not otherwise specified; PB, pancreatobiliary subtype

The OS rates for all patients at one, three, and five years were 89.3%, 67.9%, and 56.8%, respectively. The one-, three-, and five-year DFS rates were 85.3%, 65.3%, and 61.1%, respectively (data not shown). Based on the IHC classification, we analyzed the DFS in patients with AoV cancer with different subtypes (Fig. 1). The results revealed no statistically significant difference in DFS between subtypes according to classification I (*P* = 0.23). However, classifications II and III exhibited significantly different DFS among patients with different subtypes (*P* = 0.039 and *P* = 0.032, respectively). In particular, classification III showed the most differences between the four subtypes. No significant difference was found in OS (*P* = 0.092 and *P* = 0.15, respectively; data not shown). Notably, classification III indicated that patients with the INT subtype had the longest survival time, whereas patients with the PB or NOS subtypes had considerably shorter survival times (*P* = 0.017 and *P* = 0.019, respectively; Table 2). Patients with the MIX subtype had intermediate survival times. Similar results were observed for classification II (data not shown).

**Table 2:** Multiple comparison of disease-free survival according to AoV cancer subtype (classification III)

	INT	MIX	NOS
MIX	0.25		
NOS	0.019*	0.099	
PB	0.017*	0.77	0.078

*INT* Intestinal subtype, *MIX* Mixed subtype, *NOS* Not otherwise specified, *PB* Pancreatobiliary subtype

^*^P-value < 0.05

### Prognostic value of AoV cancer subtype for recurrence

The Cox proportional hazards regression model was used to analyze the prognostic factors associated with recurrence, including subtypes based on classification III (Table 3). In the univariate analysis, advanced T stage (hazards ratio [HR] = 2.141, 95% confidence interval [CI]: 1.39–3.31, *P* < 0.001), N stage (HR = 2.494, 95% CI: 1.52–4.10, *P* < 0.001), poor differentiation (HR = 2.808, 95% CI: 1.31–6.01, *P* = 0.008), and LVI (HR = 4.298, 95% CI: 2.08–8.89, *P* < 0.001) were all correlated with recurrence risk. Moreover, the PB and NOS subtypes demonstrated a higher risk of recurrence (HR = 7.753, 95% CI: 1.04–57.73, *P* = 0.046 and HR = 8.969, 95% CI: 1.04–74.55, *P* = 0.042, respectively) compared with the INT subtype. However, these factors did not show prognostic significance for recurrence in the multivariate analysis.

**Table 3:** Univariate and multivariate analyses of prognostic factors for disease-free survival

	Univariate		Mutivariate	
	HR (95% CI)	*P*-value	HR (95% CI)	*P*-value
Sex (male)	1.099 (0.54–2.24)	0.794		
Age	0.9997 (0.96–1.04)	0.988		
Tumor size	1.012 (0.80–1.28)	0.92		
T stage	2.141 (1.39–3.31)	< 0.001***	1.429 (0.83–2.46)	0.196
N stage	2.494 (1.52–4.10)	< 0.001***	1.185(0.58–2.44)	0.645
Differentiation (Poor)	2.808 (1.31–6.01)	0.008**	1.142 (0.46–2.85)	0.776
LVI (Positive)	4.298 (2.08–8.89)	< 0.001***	2.402 (0.95–6.10)	0.065
PNI (Positive)	2.025 (1.01–4.08)	0.049*	0.950 (0.40–2.24)	0.907
Subtype (Classification III)				
INT	1		1	
MIX	3.271 (0.382–28.00)	0.279	3.128 (0.37–26.23)	0.351
PB	7.753 (1.041–57.73)	0.046*	4.289 (0.56–32.94)	0.153
NOS	8.969 (1.041–74.55)	0.042*	5.22 (0.58–46.84)	0.138

*LVI* Lymphovascular invasion, *PNI* Perineural invasion, *INT* Intestinal subtype, *MIX* Mixed subtype, *NOS* Not otherwise specified, *PB* Pancreatobiliary subtype, *HR* Hazard ratio, *CI* Confidence interval

^*^P-value < 0.05; **P < 0.01; ***P < 0.001

### Clinicopathological characteristics of AoV cancer patients according to subtype

Table 4 shows the clinicopathological characteristics of patients with AoV cancer of different subtypes based on classification III. Significant differences were observed in the T stage, N stage, and LVI among the subtypes (*P* = 0.021, *P* = 0.003, and *P* = 0.045, respectively). Patients with PB-type cancer exhibited more advanced T and N stages and a higher incidence of LVI than those with other subtypes. However, no significant correlations were found between the subtypes and age, sex, tumor size, tumor differentiation, or perineural invasion (PNI).

**Table 4:** Clinicopathological characteristics of patients with AoV cancer according to subtype (classification III)

	INT	MIX	NOS	PB	*P*-value
	(*N* = 10)	(*N* = 18)	(*N* = 9)	(*N* = 38)	
Sex					0.236
F	3	5	6	14	
M	7	13	3	24	
Age	61.8 ± 9.5	62.3 ± 10.1	64.7 ± 6.8	64.8 ± 9.7	0.316
Tumor size	2.6 [2.0;5.2]	2.1 [1.9;3.0]	2.5 [1.2;3.5]	2.4 [1.8;3.0]	0.712
Differentiation					0.15
Well, moderate	10	16	7	27	
Poor	0	2	2	11	
T stage					0.021*
1	7	5	2	9	
2	1	6	1	7	
3	2	7	4	21	
4	0	0	2	1	
N stage					0.003**
0	9	15	5	19	
1	1	2	1	17	
2	0	1	3	2	
LVI					0.045*
Negative	9	15	6	20	
Positive	1	3	3	18	
PNI					0.145
Negative	9	11	8	23	
Positive	1	7	1	15	

*INT* Intestinal subtype, *MIX* Mixed subtype, *NOS* Not otherwise specified, *PB* Pancreatobiliary subtype, *LVI* Lymphovascular invasion, *PNI* Perineural invasion

^*^P-value < 0.05; **P < 0.01

## Discussion

IHC is a valuable tool for identifying the subtypes of AoV cancer based on the expression of specific proteins. In conjunction with hematoxylin and eosin staining for histopathological examination, IHC staining provides molecular information that improves the accuracy of tumor subtyping [15, 17]. By integrating multiple markers, a comprehensive and precise assessment of the tumor subtype is possible, which aids in predicting prognosis and plays a crucial role in determining the most suitable treatment strategy for individuals diagnosed with AoV cancer.

CK7, CK20, and CDX2 are IHC markers that can reliably distinguish between INT and PB subtypes [13, 15, 17, 18]. INT-subtype adenocarcinomas are CK7-negative and CK20-positive, whereas PB-subtype adenocarcinomas are CK7-positive and CK20-negative, similar to the pancreatic ductal epithelium. CDX2, a transcription factor encoded by the *CDX2* gene, is a more robust and specific marker of the INT subtype and has gained increasing interest [19, 20]. Therefore, CK20 and CDX2 expression helps to define the INT subtype. However, as AoV cancer can exhibit PB and INT characteristics, IHC staining may also show a combination of these characteristics. Although CK7 is expressed in most PB subtypes, less than 50% express CK20, a distinguishing feature of the INT subtype. Thus, the PB subtypes may not be uniquely distinguished.

In the present study, among the cases in which CK7 was negative (19 cases), 36.8% (7 cases) displayed positive CK20 expression, and 52.6% (10 cases) exhibited positive CDX2 expression. Of the 56 CK7-positive cases, 23.2% (13 cases) were CK20-positive, and 32.1% (18 cases) were CDX2-positive. When CK20 was positive, concurrent positive expression of CDX2 was observed in all but one case, highlighting a strong association between CK20 and CDX2 expression. Consequently, CDX2 was observed in 19 of the 20 CK20 + cases and 9 of the 55 CK20- cases. These findings imply that CDX2 may help determine the presence of INT characteristics, especially in CK20-negative cases.

In the present study, when cases classified as CK7-/CK20 + were considered the INT subtype (7/75, 9.3%), named classification I, there was no significant difference in DFS among the subtypes. However, when the subtypes were classified using CDX2 expression, the INT subtype was identified in 13.3% (10/75) of cases in classifications II and III, and DFS exhibited an advantage over OS. In addition, classification III, based on CK7 and CDX2 expression and not CK20 expression, showed the most significant differences among the four subtypes. AoV cancer cases with characteristics of the INT subtype (CK7-/CDX2 +) had a better prognosis than those with the PB subtype. Conversely, cases without these characteristics (CK7-/CDX2-) exhibited a worse prognosis than those with the PB subtype. Additionally, several prognostic factors for recurrence were identified, including PB and NOS subtypes based on classification III, T and N stages, LVI, and PNI; however, these factors were not independent. Furthermore, the PB subtype was associated with higher T and N stages and the presence of LVI, which is consistent with the results of previous studies [6–8]. These results indicate that subtype classification based on CK7 and CDX2 expression can influence the prognosis of patients with AoV cancer and that the clinical characteristics of the subtypes differ significantly.

CDX2 has been proposed as a highly sensitive and specific marker for adenocarcinomas of intestinal origin [20]. In the present study, employing CDX2 allowed for more accurate differentiation with respect to prognosis, compared with using CK20. Kumari et al. and Hansel et al. have demonstrated a correlation between histological subtype and survival and the expression of CDX2 as an independent marker of outcome [17, 19]. Patients with CDX-positive cancers exhibit significantly longer survival times than those with CDX-negative cancers. Adding CDX2 to the panel of immunohistochemical markers (CK7 and CK20) can increase the accuracy of subtyping, contributing to the standardization of pathology reporting. Palmeri et al. revealed that CK7, CK20, and CDX2 were sufficient IHC markers to classify AoV cancers [21]. Thus, our findings are consistent with those of prior research, providing further support and validation of existing knowledge.

There are discrepancies in the reported proportions of AoV cancer subtypes among different studies. Reid et al. classified ampullary carcinomas into four subtypes based on morphological features: mixed (40%), pure PB (32%), other (22%), and pure INT (6%) [22]. In the present study, we classified cases as INT (10/75, 13.3%), mixed (19/75, 25.3%), PB (37/75, 49.3%), and others (9/75, 12%) based solely on IHC results. The mixed and other types were more common in the study by Reid et al. than in other studies based on IHC, including ours [15, 23, 24]. Ang et al. and Al Abbas et al. reported 18% and 20% of tumors had an ambiguous phenotype, respectively [15, 24]. Although this may have resulted from the overlapping histopathological features and lack of consistent diagnostic criteria [20], previous studies emphasized the value of IHC, particularly in the less common subtypes and in tumors that are difficult to classify morphologically [15]. Therefore, compared with histopathological parameters, IHC can provide more objective and reproducible results, insights into tumor biology, and aid in the development of targeted therapies.

It has been reported that PB type is an unfavorable prognostic factor of AoV cancer [9–12]. Some studies have also examined the prognosis of different subtype based on IHC, and PB type has been reported as having a poor prognosis, consistent with our findings. [20, 24]. However, the prognostic value of the NOS type remains unexplored. Interestingly, the present study revealed that the NOS type showed the poorest DFS among all AoV subtypes. In addition, patients at T3 and N2 stage had the highest proportion of NOS type. These results imply a potential association between the NOS type and aggressive tumor biology.

Our analysis specifically focused on the DFS of patients based on their AoV cancer subtypes, unlike the OS analyses in other studies. OS analysis, which includes deaths from non-disease-related causes, could potentially confound our results. Therefore, in the present study, we assessed the duration for which patients remained free from cancer recurrence, providing insights into the potential impact of classification on disease progression and treatment effectiveness. Recently, the approaches of administering gemcitabine-based therapies for PB-type tumors and fluorouracil-based therapies for INT-type tumors have been increasingly favored [24–26]. However, the present study’s retrospective nature made further analysis of the relationship between subtypes and chemotherapy impossible. Additionally, our study is limited by the small sample size and reliance on data from a single center and a single surgeon. Nevertheless, this study suggests that classifying AoV cancer subtypes using only IHC results has clinical significance. Further studies are needed to validate the results of this study and confirm the characteristics of the NOS and MIX subtypes.

## Conclusions

In conclusion, classification using CDX2 expression revealed distinct subtypes with prognostic significance, indicating that CDX2 is an essential marker for distinguishing INT characteristics. Therefore, combining CK7 and CDX2 or adding CDX2 to CK7/CK20 allows for a more comprehensive evaluation of the AoV cancer subtype and aids in predicting prognosis.

### Supplementary Information

**Additional file 1:** **Supplementary Figure 1.** Representative images of immunohistochemical staining for classification of AoV cancer subtype. Intestinal (INT), mixed (MIX), pancreatobiliary (PB), and not otherwise specified (NOS) subtypes showed distinct expression of CK7, CK20, and CDX2. Images of hematoxylin-eosin-stained tissues were also represented. Magnification 200×.

## Data Availability

The datasets used in the present study are available from the corresponding author on reasonable request.

## Abbreviations

AoV: Ampullar of Vater
CDX2: Causal-type homeobox transcription factor 2
CK7: Cytokeratin 7
CK20: Cytokeratin 20
IHC: Immunohistochemical
INT: Intestinal subtype
MIX: Mixed subtype
NOS: Not otherwise specified
PB: Pancreatobiliary subtype
