# Supplementary material for: Clinical impact of ampulla of Vater cancer subtype classification based on immunohistochemical staining
Source: World J Surg Oncol. 2024 Jan 3;22:5. doi: 10.1186/s12957-023-03289-y (PMC10763163; doi:10.1186/s12957-023-03289-y)
Supplement: Supplementary file 1 — Additional file 1: Supplementary Figure 1. Representative images of immunohistochemical staining for classification of AoV cancer subtype. Intestinal (INT), mixed (MIX), pancreatobiliary (PB), and not otherwise specified (NOS) subtypes showed distinct expression of CK7, CK20, and CDX2. Images of hematoxylin-eosin-stained tissues were also represented. Magnification 200×. [file 12957_2023_3289_MOESM1_ESM.docx]

**Supplementary Material**

**
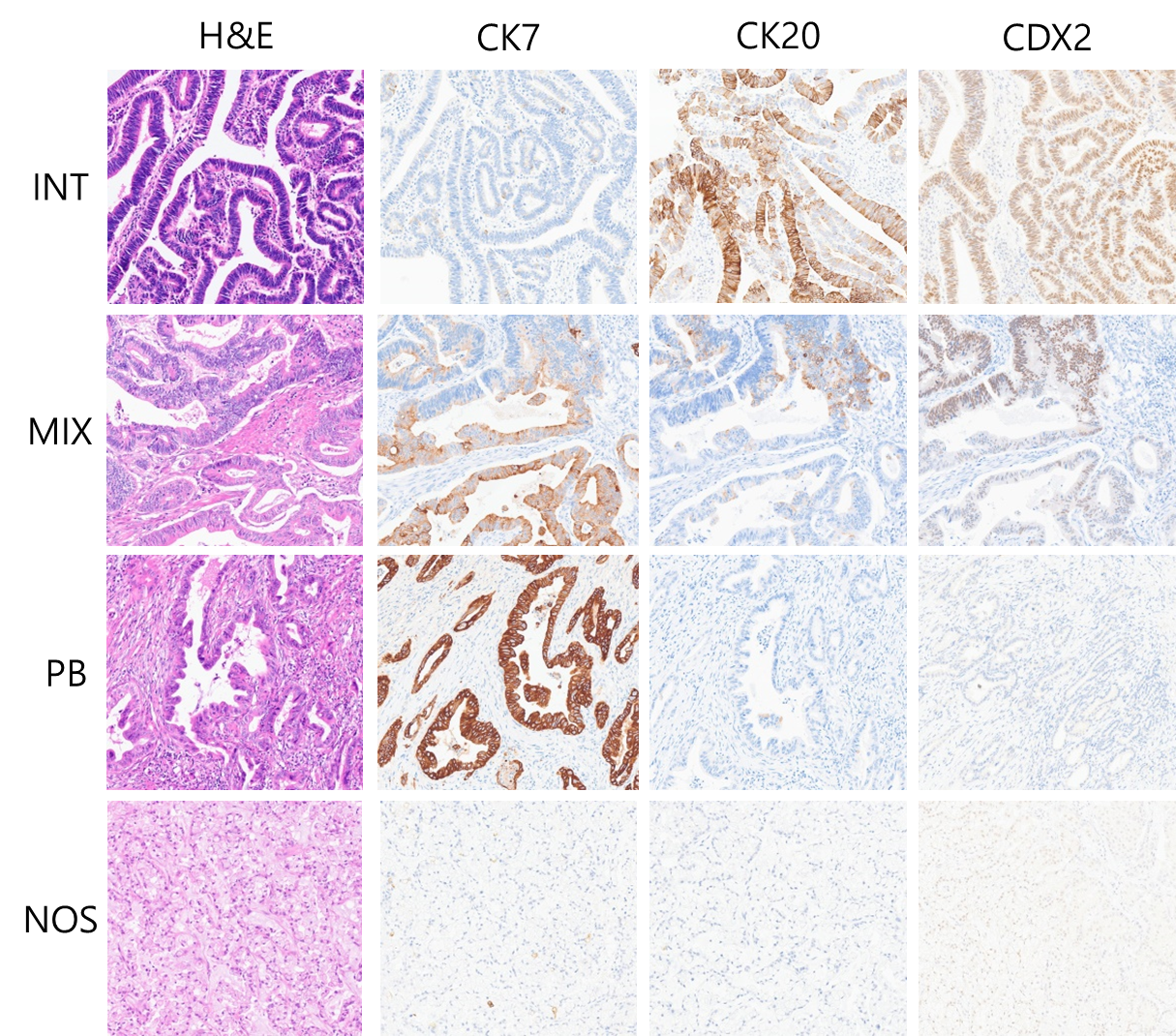
**

Supplementary Figure 1. Representative images of immunohistochemical staining for classification of AoV cancer subtype. Intestinal (INT), mixed (MIX), pancreatobiliary (PB), and not otherwise specified (NOS) subtypes showed distinct expression of CK7, CK20, and CDX2. Images of hematoxylin-eosin-stained tissues were also represented. Magnification 200×.
